# Testing for Functional Adaptation in a Genital Bone: Baculum Size, Shape, and Microstructure Show Little Plastic Response to Mating Across Five Rodent Species

**DOI:** 10.1093/iob/obag043

**Published:** 2026-09-02

**Authors:** C F Toney III, N G Schultz, B Pomidor, J Taylor, J Andronowski, M D Dean

**Affiliations:** Molecular and Cellular Biosciences, University of Southern California, USC 90089, Los Angeles, USA; Molecular and Cellular Biosciences, University of Southern California, USC 90089, Los Angeles, USA; Molecular and Cellular Biosciences, University of Southern California, USC 90089, Los Angeles, USA; Division of BioMedical Sciences, Faculty of Medicine, Memorial University of Newfoundland, A1C 5S7, St. Johns, Canada; Division of BioMedical Sciences, Faculty of Medicine, Memorial University of Newfoundland, A1C 5S7, St. Johns, Canada; Molecular and Cellular Biosciences, University of Southern California, USC 90089, Los Angeles, USA

## Abstract

Plasticity—the ability of a genotype to produce different phenotypes in response to environmental input—can provide insight into the mechanical forces experienced by biological structures. Bones are particularly plastic, remodeling in response to sustained and repetitive mechanical stress. In many mammals, males possess a bone in their penis called a baculum. Across species, its extreme morphological divergence suggests it undergoes recurrent adaptive evolution, and several studies have explicitly linked baculum size and shape to reproductive fitness. However, the precise functions of this bone remain largely unknown. Here we tested whether copulation imposes mechanical loading sufficient to induce remodeling in the baculum. We compared bacula of mated vs. unmated males across five rodent species with divergent baculum morphologies and mating ecologies, using high-resolution morphometric analyses of size and shape, and synchrotron-enabled quantification of bone microstructure. We found no consistent effect of mating on size, shape, or microstructure of the baculum. These results suggest that copulation does not impose mechanical loads sufficient to induce remodeling in the baculum. More broadly, our findings indicate that the rapid evolutionary divergence of the baculum is unlikely to be enabled by plasticity associated with copulation.

## Introduction

Almost universally, genitalia evolve rapidly, leading to substantial and seemingly disproportionate interspecific divergence (Klaczko *et al.* 2015). Male genitalia vary not only in overall size and shape, but also in the occurrence and morphology of accessory structures such as ridges, flanges, and penile spines (Eberhard 1985; Brennan and Prum 2015; Klaczko *et al.* 2015; Orr and Brennan 2016; Keeffe *et al.* 2025). Among mammals, many species also possess a baculum, an ossified structure that resides in the glans penis (Burt 1960; Long and Frank 1968; Patterson 1983). Like other features of genitalia, the baculum evolves rapidly and shows an astonishing level of morphological variation between species. In addition, the baculum has also been gained and lost multiple independent times across mammal lineages (Schultz *et al.* 2016). While the precise functions of this bone are enigmatic, its morphological diversity is likely driven by sexual selection, via male-male competition, male-female conflict over reproductive outcomes (Eberhard 1985; Kinahan *et al.* 2007; André *et al.* 2018, 2022), and/or coevolutionary interactions between male and female genitalia (Brennan and Prum 2015; Orr and Brennan 2016; Andonov *et al.* 2017).

Within various mammal groups, baculum length has been shown to covary with intromission length (Dixson 1987a, 1987b; Herdina *et al.* 2015; Brassey *et al.* 2018; Clear *et al.* 2023), relative testis size (Ramm 2007; Fitzpatrick *et al.* 2012; Stockley *et al.* 2013; André *et al.* 2018), female reproductive investment (André *et al.* 2022), and measures of male quality (Miller *et al.* 1999; Miller and Burton 2001; Tasikas *et al.* 2009). However, these correlations are not consistently found across clades (Hosken *et al.* 2001; Larivière and Ferguson 2002; Lüpold *et al.* 2004; Ramm *et al.* 2010; Schulte-Hostedde *et al.* 2011; Schultz *et al.* 2016), making it difficult to conclusively assign function(s) to the baculum from comparative studies alone.

Intraspecific studies, particularly using house mice (*Mus sp.*), have hinted that the baculum may influence reproductive fitness. In semi-natural enclosures with large numbers of males and females, males with relatively wider bacula sired more litters with significantly more offspring, suggesting that baculum width may influence both paternity and female reproductive investment in potentially competitive environments (Stockley *et al.* 2013). Among wild mice, males from relatively promiscuous populations possess bacula with wider proximal segments, suggesting width increases reproductive success under competitive mating regimes (Simmons and Firman 2014). Experimental evolution directly manipulating promiscuity has confirmed that males evolving under sperm competition develop wider bacula compared to males in monogamous mating regimes, a difference shown to be genetically determined (Firman and Simmons 2008; Simmons and Firman 2014). Males with relatively wide proximal bacula also father more offspring than those with thinner bacula when in controlled mating experiments, although this advantage is responsive to both mate order effects and coevolution with the female reproductive tract (André *et al.* 2018; Winkler *et al.* 2021). Notably, under competitive mating conditions, males with relatively thin bacula are able to rescue their apparent disadvantages if they mate with a female for longer before ejaculation. (André *et al.* 2022). Taken all together, these studies suggest that the baculum is likely acting to stimulate the female reproductive tract in a manner that influences female reproductive investment, and that in house mice at least, a relatively wider proximal end is advantageous under competitive mating conditions.

Despite these associations, the precise mechanical functions performed by the baculum during intromission, influencing reproductive fitness, remain unclear. In this study, we employ a strategy complementary to those described above in order to infer function, by leveraging the potential of the baculum to respond plastically to mechanical loading. Bones remodel in response to sustained or repetitive stresses (Frost 1994; Ruff *et al.* 2006; López-Aguirre *et al.* 2021) and several features of the baculum suggest it would be particularly capable of adapting functionally in this way.

Like most load bearing elements of the mammal skeleton, the proximal baculum is physiologically active, as evidenced by incorporation of calcein over short periods of time (Schultz, 2019), and composed of endochondral bone, which is particularly responsive to mechanical cues (Mackie *et al.* 2008; Maes 2013). The proximal baculum also serves as an attachment site for corpus cavernosa and ischiocavernosus muscles that are believed to support the baculum during intromission (Kelly 2000). Consistent with this interpretation, genetically engineered mice that lack the proximal muscle attachment sites, but are otherwise phenotypically normal, often fail to copulate (Ghione *et al.* 2025). Furthermore, in rats (*Rattus norvegicus*), the baculum shows patterns of mineralization that suggest the proximal segment is load-bearing (Kelly 2000) and, although the baculum was not measured specifically, the penises of sexually experienced rats are significantly heavier than those of sexually inexperienced counterparts, suggesting the physics of intromission can affect genital development (Taylor *et al.* 1983; Kelly 2000).

Several additional features indicate that the baculum may be poised to respond plastically. It develops after birth (Ghione *et al.* 2025), as the animal begins interacting with its outside environment, and its morphology depends on androgen signaling (Ghione *et al.* 2025), which varies dramatically over an individual male’s environment and experience. Furthermore, young males exposed to the scent of potential rival males develop relatively wider bacula, consistent with a role of plasticity (André *et al.* 2018). Importantly, although these results suggest that the baculum is capable of responding plastically to environmental cues, they do not establish whether the baculum is adapting functionally to mechanical loadings during intromission.

If the baculum experiences sustained or repeated mechanical stress above a certain threshold during intromission, it should exhibit detectable remodeling within individuals over time. Conversely, the absence of such remodeling would suggest that intromission does not impose loads that consistently exceed that threshold. Because load-induced remodeling in bone typically produces coordinated changes in size, shape, and internal structure, such effects should be detectable across multiple structural scales if present.

Here we compared size, shape, and microstructure of bacula from sexually experienced versus virgin males, across five species of rodents (*Mus domesticus* [house mice], *Peromyscus maniculatus, P. leucopus, P. californicus, and P. polionotus* [deer mice]). We predicted that bacula from sexually experienced males would be more robust than bacula from virgin males, and that the direction of variation would allow us to infer the physical forces a baculum experiences during intromission (Kelly 2000; Tasikas *et al.* 2009). These five species span multiple axes of morphological and ecological variation—ranging from the long gracile bacula of *Peromyscus leucopus* to the spatulate-shaped bacula of *Mus domesticus* (Fig. 1), and from the monogamous *P. californicus* and *P. polionotus* to the promiscuous mating systems of the other three species (Foltz 1981; Ribble 1991; Xia and Millar 1991; Gubernick and Nordby 1993; MacManes 2011). These five species last shared a common ancestor ∼20–25 million years ago (Steppan *et al.* 2004; Steppan and Schenk 2017; López-Antoñanzas *et al.* 2024), further increasing our chances of identifying plasticity that could be species-specific.

**Fig. 1:**
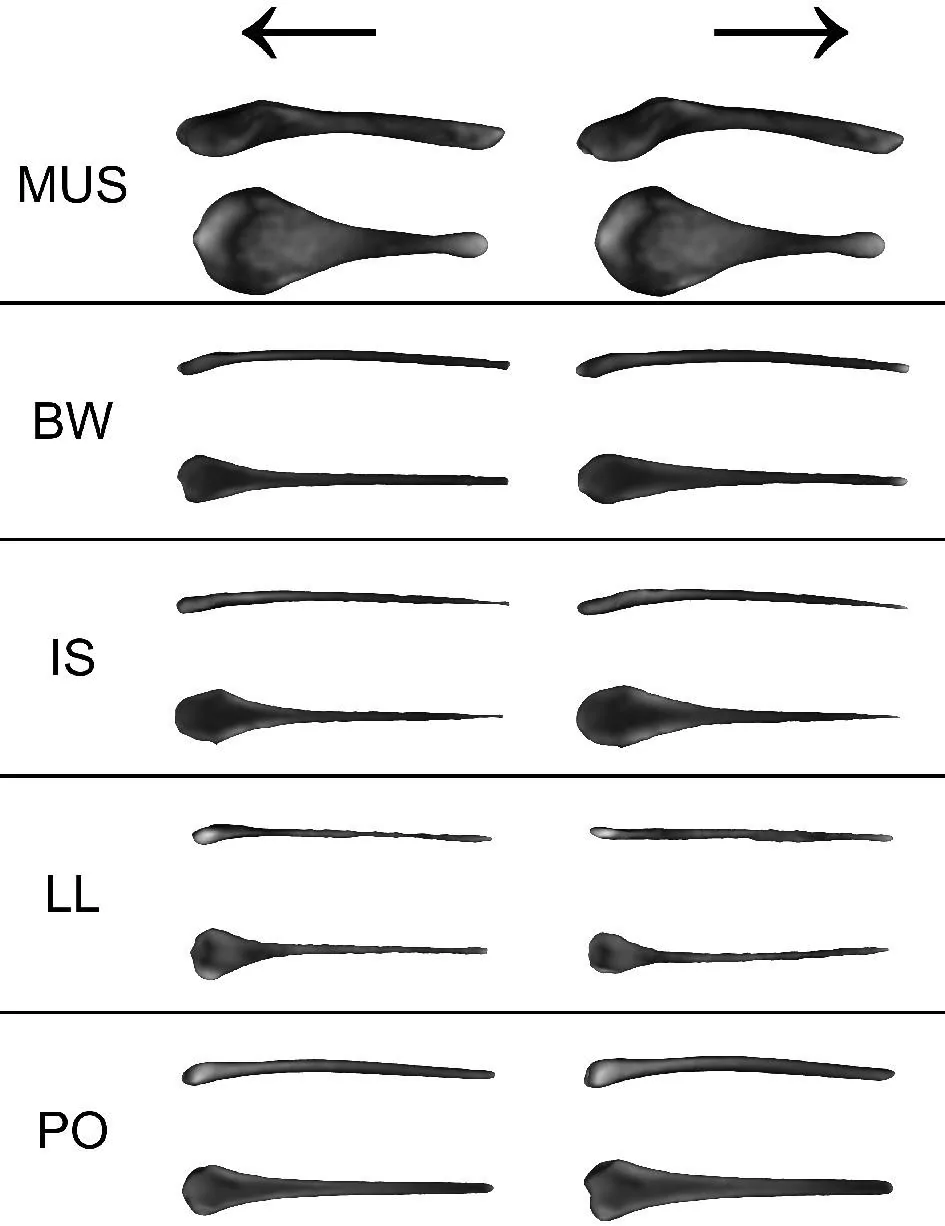
Principal Components projections of surface meshes following superimposition and homologization by GPSA2. Each “row” represents a species: MUS=*Mus musculus*, BW=*Peromyscus maniculatus bairdii*, IS=*Peromyscus californicus insignis*, LL=*Peromyscus leucopus*, PO=*Peromyscus polionotus subgriseus*. Within each species, top mesh shows lateral view, bottom mesh shows dorsal view. The left column and right columns indicate opposite-signed projections into PC1 space. MUS is projected from -3 to +3, all four *Peromyscus* species are projected from -1 to +1 to more closely mirror the observed range. Viridis colors range from dark blue to yellow, with yellow indicating regions of high variation in PC1 across samples. In all images, distal is to the right of the figure.

In house mice (*Mus domesticus*), we compared bacula of males that experienced continuous copulation over a 60 day period to bacula of unmated males, quantifying size and shape through morphometric analyses of microCT scans, and synchrotron-enabled measures of bone composition. In deer mice (four species of *Peromyscus*), we compared bacula of sexually experienced stud males to bacula of age-matched virgin males. We do not find strong or consistent evidence of plasticity, suggesting that copulation is unlikely to impose mechanical loads sufficient to induce strong remodeling in the baculum.

## Materials and methods

All experimental procedures were approved by the University of Southern California’s Institutional Animal Care and Use Committee, protocol #21218.

### House mice

Twelve 7-week-old male house mice (genus *Mus*, strain C57BL/6J) were purchased from The Jackson Laboratory (strain #000664). Males were shipped as trios. After a week of acclimation, they were divided equally into four treatments, taking care not to include more than one male from a shipping container in the same treatment. In the “mating” treatment, three males were separately housed with two females each, for a period of 60 days. All females were checked twice daily for copulatory plugs, an indication that mating has occurred (Schneider *et al.* 2016). Plugged females were immediately removed and replaced by a sexually mature virgin female. Plugged females were kept in separate cages to track pregnancies and litters born. Individual females were tracked through strategic ear-punching.

We implemented three different control treatments alongside the mating treatment. In the “individual” controls, three males were housed individually, without female or male contact. In the “triplet” control, three males were housed together. Like individual males, triplet males had no exposure to females, but their cage matched the total number of individuals in mating regime cages. In a “scent” control, three males were housed individually, but with daily introduction of soiled bedding from all-female cages. These “scent” controls were meant to maintain exposure to females, which could be important if females induce hormonal changes in males that influence baculum morphology.

We euthanized males after 60 days in each treatment, recording body mass and testes mass. We dissected their entire penises, fixed them in formalin and dehydrated them with immersion in increasing concentrations of ethanol; 70% (3 days), 80% (3 days), and 100% (3 days). These males copulated with an average of 29 females over three months (see Results).

### Deer mice

Deer mice (genus *Peromyscus*) have a baculum that is longer and thinner compared to the more stout baculum of house mice. We purchased frozen carcasses of four species—*Peromyscus maniculatus bairdii* (strain BW), *Peromyscus polionotus subgriseus* (strain PO), *Peromyscus leucopus* (strain LL) and *Peromyscus californicus insignis* (strain IS) from the Peromyscus Stock Center at the University of South Carolina, College of Pharmacy. For each species, we obtained two breeder males, each of which had been paired continuously with a female since sexual maturity and had sired between nine and eighteen separate litters—likely a significant underestimate of the true number of copulations. Breeder males were compared to age-matched virgin males. As with the house mice, we dissected their entire penises, fixed them in formalin and dehydrated them with immersion in increasing concentrations of ethanol; 70% (3 days), 80% (3 days), and 100% (3 days).

### MicroCT scanning and surface mesh extraction of the baculum

Samples were microCT-scanned by the PaleoCT Center at the University of Chicago. *Mus* penises were imaged on a Phoenix v|tome|x s 240 with a dual 180 tube (RRID:SCR_024763), with the following parameters: 15.0 uM resolution, 90 kV, 170 mA current, 200 ms timing, with 1500 projections. Parameters for *Peromyscus* penises differed only by using 150 ms timing.

We imported microCT image stacks into Slicer3d, segmented out individual bacula and extracted their outer surface mesh using the “Wrap Solidify” algorithm, with the following parameters: oversampling factor=1.5, joint sampling=0.00, decimation factor=0.00 (Fedorov *et al.* 2012). We imported all surface meshes into MeshLab (via the pymeshlab Python module) and implemented the Screened Poisson Surface Reconstruction (Kazhdan *et al.* 2006) with default parameters, followed by Isotropic Explicit Remeshing (Hoppe *et al.* 1993), also under default parameters with the exception of percentage target length, which we set to 0.5. Together, these two algorithms simplified the surface mesh with faces that better approximated equilateral triangles without losing any resolution of the surface, resulting in a cleaner model.

### Morphometric analyses

We quantified size and shape using the morphometric approaches of GPSA2 (Pomidor and Dean 2024). GPSA2 uses the intuitive framework of Generalized Procrustes Analyses to produce homologized surface meshes, which are surface meshes that each only contain faces with the best reciprocal matches across the sample and are aligned as closely as possible. GPSA2 iteratively homologizes and superimposes surface meshes by minimizing the differences between three main sources of information: direction of normals, position of faces in xyz space, and local shape descriptors. Although GPSA2 is a hybrid approach that combines landmark-free approaches with landmarks, visual inspection showed excellent superimposition without landmarks. Therefore, we ran GPSA2 in an entirely landmark-free framework, but analyzed each species separately to avoid interspecific variation swamping out intraspecific variation.

To quantify shape differences, we employed Principal Components Analysis on the xyz coordinates of all vertices from the homologized surface meshes. Any Principal Components that explained at least 5% of variance were analyzed in a permutational multivariate analysis of variance (PERMANOVA), using the ADONIS2 function of the R package VEGAN (Oksanen *et al.* 2026), using Euclidean distance and 999 permutations to test the influence of experimental treatment on baculum shape. We also compared size among treatments, computed in GPSA2 as the surface area-weighted centroid size (Pomidor and Dean 2024). The area-weighted centroid size is the distance from every vertex to the centroid (average x, y, z), weighted by the average area of connected faces.

### Synchrotron radiation-based micro-CT

For house mice from the “mating” and “individual” treatments only (N=6 total) we also gathered synchrotron data to investigate variation in bone microstructure associated with mating. Preserved penises from these males were rehydrated in warm water and bacula were dissected out for synchrotron analysis. Bacula were imaged at the Canadian Light Source in Saskatoon, Saskatchewan, on the BioMedical and Imaging Therapy Bend-Magnet Beamline (BMIT-BM). The white beam microscope was equipped with 0.883 mm aluminum and 0.084 mm molybdenum filters and a 5x objective lens, resulting in an effective pixel size of 1.5 µm. The setup included a PCO Edge camera. Additional experimental parameters included a 20 keV energy level, exposure of 150 ms, 180° rotation, and a step angle of 0.06, resulting in 3,000 projections/sample.

The projectional data were reconstructed using the tofu image processing toolkit and microstructural features of interest (e.g., pores) segmentated via ORS Dragonfly (Faragó *et al.* 2022). Twelve different phenotypes were gleaned from the synchrotron datasets such as pore surface area, pore separation, pore surface area/volume ratio, pore number, pore diameter, pore volume, and overall porosity. We also performed Principal Components Analysis on these 12 phenotypes using the *prcomp* function in R, with scale=TRUE and center=TRUE. Together, these analyses focused on structural differences at extremely high resolution.

### Re-analysis of previously published mouse bacula

We re-analyzed baculum data from Schultz (2019), which included N=14 mated and N=7 unmated males. In that study, males classified as “mated” were paired continuously with one novel sexually mature female per week, for a period of 7–9 weeks, whereas unmated males were paired together with no females. We analyzed those surface meshes using the same GPSA2 morphometric pipeline described above to allow direct comparison with our primary dataset. All surface meshes are included in the Dryad archive associated with this manuscript: https://doi.org/10.5061/dryad.jm63xsjrp.

## Results

### Mating trials

The three “Mating” males copulated with 23, 29, and 35 females, beginning 2–4 days after the experiment commenced and extending over the 60-day period. Copulation occurred every 2.13 days on average. Of these copulations, ∼34% resulted in offspring. This is a relatively low reproductive success (Dean 2013), which could be at least partially explained by the removal of females after only one copulation, or by the fact that virgin females often lose their first litter (Silver 1995).

### Baculum size, shape, and composition are largely unaffected by mating history

Following GPSA2, homologized and superimposed surface meshes of house mouse bacula consisted of 19,188 vertices and 38,372 triangular faces (Fig. 1). In *Mus*, PC’s 1 through 6 accounted for 34.3%, 17.7%, 12.0%, 8.4%, 5.6%, and 5.4% of the variance in shape, respectively. Variation in PC1 ranged from a relatively narrow and flat baculum, to a more curved baculum with a wider proximal half (Fig. 1). Visual inspection of PC1 and PC2 did not show strong separation among the treatments (Fig. 2).

**Fig. 2:**
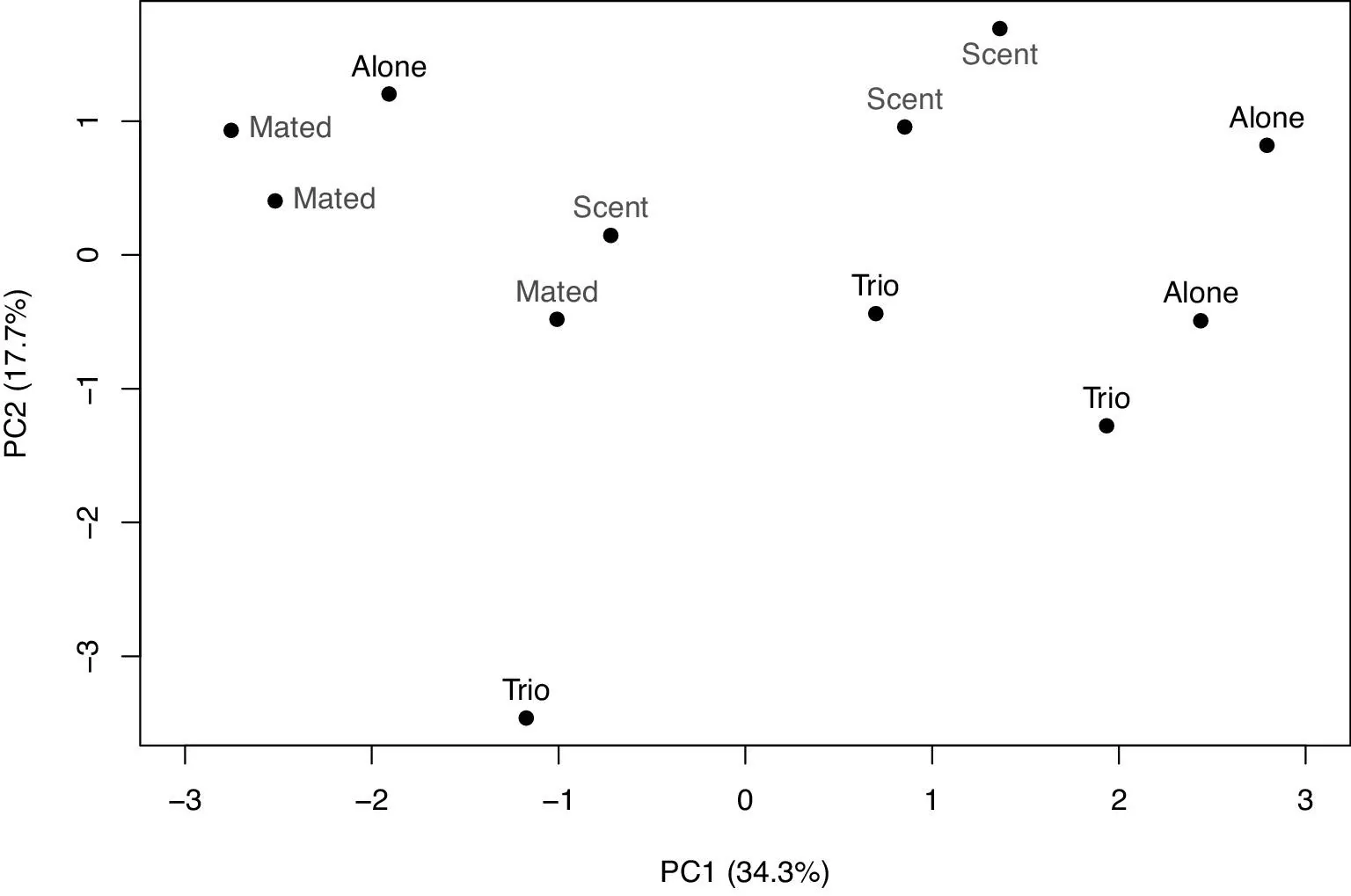
*Mus* bacula projected into PC space. Mated=males under continuous mating. Alone=singly housed males. Scent=singly housed males exposed to soiled female bedding. Trio=three males housed together.

PERMANOVA uncovered inconsistent results. We found no difference among the four MUS treatments (F_3,8_=1.6, p=0.096, Table 1), but if we pooled all unmated males together, a modest shape difference between mated and unmated males was detected (F_1,10_=2.8, p=0.028) (Table 1). This latter result holds regardless of how many PC’s we include in the PERMANOVA, but the former result is sensitive to PC inclusion. We detected shape differences between the four treatments only when we analyzed PC1-3 (F_3,8_=2.3, p=0.045, Fig. 3). Mated males tended to have a negative PC1 (left arrow in Fig. 1), which describes a flatter, less robust baculum—opposite what is predicted by bone remodeling theory (Ruff *et al.* 2006; Simmons and Firman 2014; André *et al.* 2018). We also tested each PC separately in univariate linear models: PC1 described significant variation in shape between mated and unmated *Mus* males, but only after pooling the three unmated controls together (t.test p=0.01, Table 1). No other univariate comparisons detected shape difference between mated or unmated bacula. Centroid size did not differ among treatments (Table 1).

**Fig. 3:**
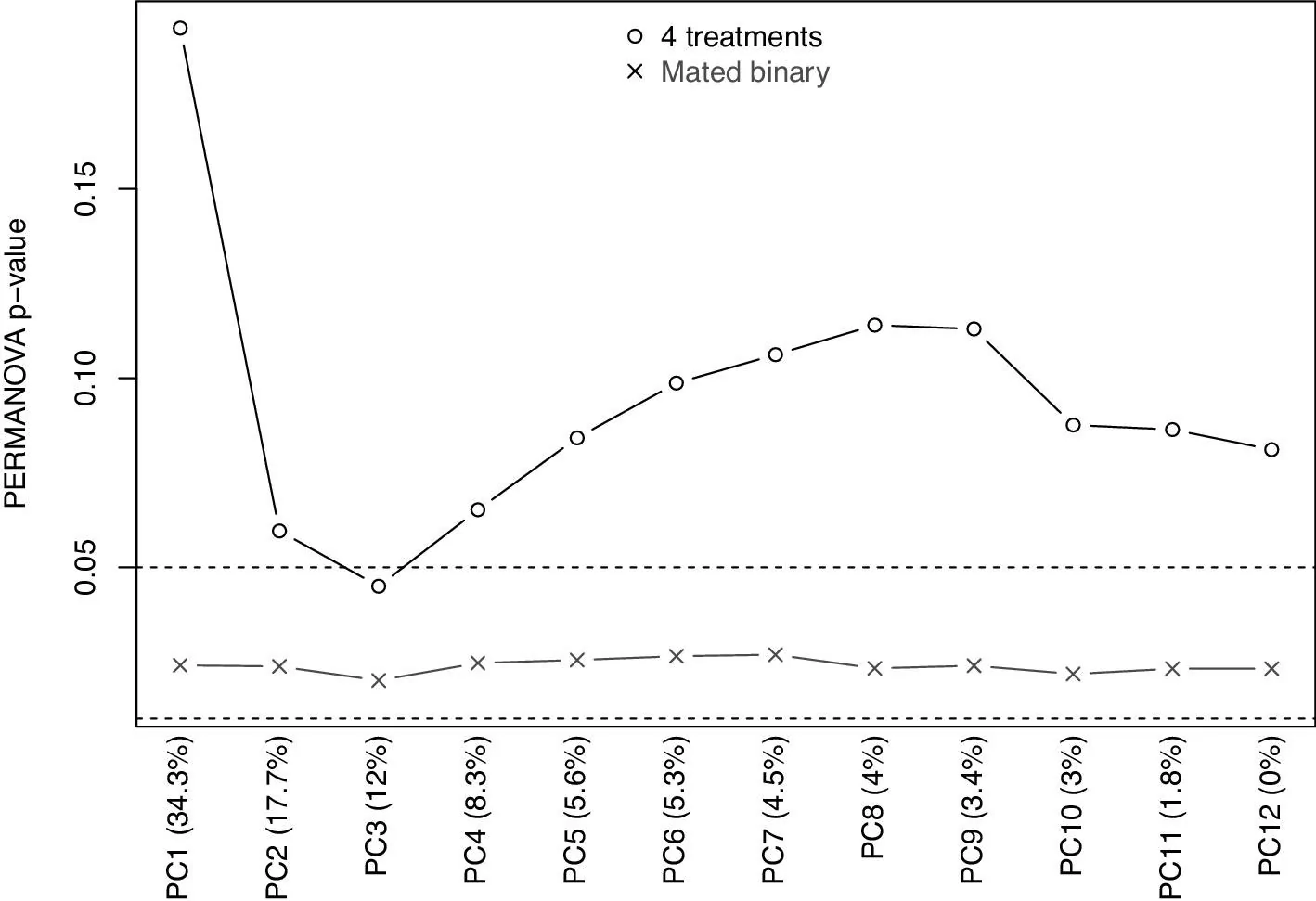
Sensitivity of PERMANOVA results to inclusion of PC’s, under two different analytical regimes: one testing for differences between all four treatments (4 treatments) and one where the three unmated male treatments are pooled (Mated binary). *Mus* bacula do not differ across the four treatments, except when PC1 through PC3 are included in PERMANOVA. When *Mus* bacula are binarized into mated vs. ummated, a shape difference is detected under PERMANOVA, regardless of how many PC’s are included.

**Table 1:** *P* values from various comparisons. Multivariate for *Mus* includes PC1-6. Multivariate for the first three *Peromyscus* species includes PC1-3, and PC1-2 for *P. polionotus*.

		Univariate				
	Multivariate	PC1	PC2	PC3	PC4-PC6	Centroid size
*Mus* (4 treatments)	0.096	0.17	0.06	0.35	>0.76	0.84
*Mus* (Binary)	**0.028	**0.01	0.59	0.17	>0.42	0.82
*Peromyscus* (BW)	0.330	0.33	0.45	0.93	-	0.47
*Peromyscus* (IS)	0.670	0.65	0.27	0.59	-	0.94
*Peromyscus* (LL)	1.000	0.99	0.35	0.40	-	0.13
*Peromyscus* (PO)	1.000	0.73	0.51	-	-	0.79

Across four *Peromyscus* species (N=4 bacula each), we detected no differences in shape related to mating status. For BW, IS, LL, and PO, homologized meshes consisted of 1,911 (3,818), 1,679 (3,354), 1,421 (2,838), and 2,887 (5,764) vertices (faces), respectively (Fig. 1). PERMANOVA revealed no difference among the four *Peromyscus* species (Fig. 4, Table 1). PC1 explained 73%, 43%, 79%, and 81% of the variance in BW, LL, IS, and PO, respectively, describing variation that ranged from a proportionately smaller proximal bulb to a relatively large one (Fig. 1). PC2 explained 18%, 16%, 13%, and 30% of shape variance across the four *Peromyscus* species, respectively. Centroid size did not differ among treatments (Table 1).

**Fig. 4:**
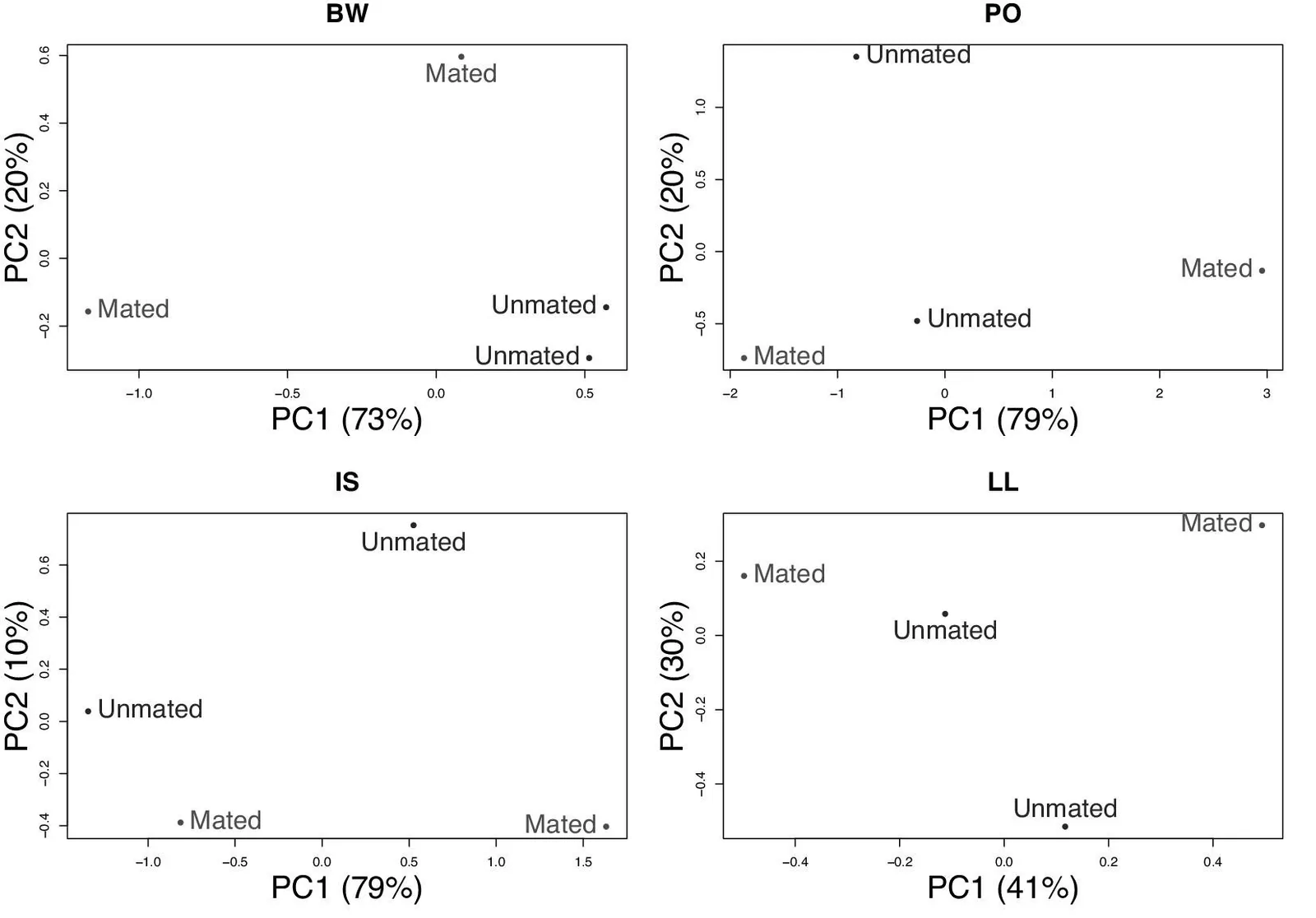
*Peromyscus* bacula projected into their species-specific PC space. BW=Peromyscus maniculatus bairdii, IS=Peromyscus californicus insignis, LL=Peromyscus leucopus, PO=Peromyscus polionotus subgriseus. For all four species, baculum shape does not significantly vary according to mating status.

### Microarchitecture unaffected by mating history

At the scale of SRµCT, which is sufficient to measure cortical porosity, *Mus* bacula did not differ among experimental groups. Of 12 phenotypes tested, plus four of their Principal Components (explaining 45.8%, 27.6%, 18.1%, and 7.6% of the variance in the 12 phenotypes, respectively), the minimum uncorrected p value calculated was p=0.065 (Table 2). Thus, we found no evidence that mating history affected cortical microarchitecture.

**Table 2:** Synchroton phenotypes compared between "mating" and "single" treatments.

Parameter	Description	*t*-test_*P* value
Bone surface area	The total surface of all porous structures calculated via the marching cubes model.	0.8814
Bone volume	The total volume of the porous structures that is calculated using the marching cubes model.	0.2554
Even effect of load on bone unitless.	The eveness of which the bone experiences loading in different directions (0=uneven, 1=even).	0.2713
Feret max µm.	The distance between the two farthest points across a given plane.	0.7055
Feret min µm.	The distance between the two closest points.	0.2131
Number of pores	The number of identifiable, distinct, and labeled porous structures thoughout the sample.	0.5452
Percent bone volume	The volume of bone per total volume analyzed.	0.2462
Percent pore volume	The volume of all pores per total volume analyzed.	0.2462
Polar moment of inertia µm^4^.	Polar moment of inertia is a quantification of resistance to bending forces around a given axis.	0.762
Pore separation	The diameter of the largest sphere that fits in the space between the structures.	0.3423
Pore surface area	The total surface of all porous structures calculated via the marching cubes model.	0.6808
Pore surface area/volume ratio	A ratio of the surface area to the volume of all structures.	0.4691
Pore thickness	The diameter of the largest sphere that fits within the structure.	0.5878
Pore volume	The total volume of the porous structures that is calculated using the marching cubes model.	0.4079
The.symmetric effect of torsion on bones	This ratio indicates the eveness of torsional load on bone, which reflects its torsional resistance.	0.3567
PC1 (45.8%)	Principal componets analysis, PC1, of univariate phenotypes.	0.3152
PC2 (27.6%)	Principal componets analysis, PC2, of univariate phenotypes.	0.6069
PC3 (18.1%)	Principal componets analysis, PC3, of univariate phenotypes.	0.726
PC4 (7.6%)	Principal componets analysis, PC4, of univariate phenotypes.	0.0655

### Reanalysis of previously published data reveals no effect of mating history

Given the variable signal observed in *Mus*, we re-analyzed baculum data from Schultz (2019) to increase sample size and assess robustness of our findings. Consistent with our primary analyses, we detected no differences in baculum shape between mated and unmated males (PERMANOVA F_1,19_=1.27, p=0.21) and no difference in size (t=1.22, df = 19, p = 0.238). Thus, across independent datasets and larger sample sizes, we find no evidence that mating history influences baculum morphology.

## Discussion

Many comparative studies have attempted to shed light on baculum function by testing for correlations between morphological features and aspects of mating ecology. These studies vary widely in their conclusions (Schultz *et al.* 2016) and the function of this unusual genital bone remains enigmatic. Rather than rely on cross-species correlations, we tested whether baculum morphology varied according to a male’s mating history, with the logic that differences between the bacula of sexually experienced and virgin males could be used to infer how the baculum is stressed during intromission. This is an important step towards understanding its interaction and perhaps co-evolution with the female reproductive tract.

We predicted that the baculum would show evidence of remodeling in response to mating history. Bone remodeling depends on the magnitude, frequency, and consistency of mechanical stress, and it is not clear whether the physics of intromission are sufficient to trigger strong remodeling in the baculum.

Across five species and multiple structural scales, we found no consistent evidence that mating history induces strong baculum remodeling, suggesting that intromission does not generate mechanical loads sufficient to trigger adaptive bone responses. This suggests that the baculum is not routinely subjected to sustained mechanical loads sufficient to induce adaptive remodeling, as observed in other load-bearing bones. Although we detected a modest difference in the shape of *Mus* bacula with respect to mating history, this emerged only after pooling all unmated males together, and the direction of shape change was toward a flatter, less robust morphology in PC1 space, and it did not correlate with any microstructural variation. This pattern is opposite to predictions, where load-induced remodeling is expected to increase mass of the proximal region of the baculum. We also found no evidence of plasticity with respect to mating history across four *Peromyscus* species despite their more gracile architectures and variation in natural mating ecologies. If intromission imposed loads sufficient to induce remodeling, we would expect concerted changes in bacula, including localized thickening, shifts in mineral density, or some asymmetry reflecting patterns of strain.

The absence of such changes suggests that intromittent stresses fall below thresholds that trigger remodeling, or are dispersed from the baculum to the soft tissues of the penis, such as the corpus cavernosa. The latter interpretation has some support: in rats the penises of sexually experienced males are heavier than those of inexperienced males (Kelly 2000). This increased weight could reflect greater robustness of supportive soft tissue within the penis, dispersing any loads that would otherwise be directed towards the baculum.

Although the baculum is morphologically similar to other load-bearing bones in mammals, intromission is far less frequent than other behaviors like locomotion or mastication that are known to induce remodeling, and also likely less physically stressful (Lad *et al.* 2019). Perhaps post hoc remodeling is not an adaptive strategy to maximize reproductive fitness. Selection may favor durability and readiness for mating opportunities rather than plasticity. In this sense, the baculum may resemble other specialized skeletal elements such as bovid horns (Davis *et al.* 2014) that prioritize performance over metabolic flexibility, preserving functionality even when unused for extended periods.

It is possible that more subtle evidence of plasticity could have been detected with larger sample sizes. However, convergent evidence across five species, as well as re-analysis of Schultz (2019), together with detailed analyses of Mus microarchitecture, supports the same conclusion: copulation does not appear to induce substantial remodeling.

Previous studies have shown the baculum develops plastically in response to perceived sperm competition risk (André *et al.* 2018), which is common in nature (Dean *et al.* 2006; Firman and Simmons 2008). In their experiment, Andre *et al.* compared “risk” males (housed individually but in tubs containing three cages where males exchanged scents and other interactions) vs. “no-risk” males (housed individually in tubs with no other individuals). Risk males developed a baculum with a broader proximal end. What might explain this difference? The exposures of Andre *et al.* spanned 21–90 days of age, almost identical to our exposures of 30–90 days. Andre *et al.* studied wild-derived house mice, which harbor ample genetic variation, unlike the classical inbred strain C57BL/6J we used here. Perhaps plasticity has been lost during inbreeding and laboratory maintenance—however, we included four different species of *Peromyscus* that also did not show strong evidence of plasticity. These results suggest that plasticity may be induced by variable social contexts, but unlikely to be primarily induced by mechanical loading. It is also important to recognize that risk males were much larger than no-risk males, and it is possible that shape variation is correlated to body size variation, rather than perceived risk of sperm competition *per se* (Tasikas *et al.* 2009).

Our main goal was to leverage predictions of bone remodeling to potentially infer physical forces experienced during intromission and copulation. However, our results speak to a larger theoretical connection between plasticity and evolution that has long interested biologists. It has been proposed that phenotypic plasticity may be an important enabler of adaptive evolution, by allowing traits to “explore” various optima before canalization by selection and genetic assimilation (Simpson 1953; Waddington 1953, 1959; Pfennig 1992; West-Eberhard 2003; Lande 2009; Bittner *et al.* 2021; Baldwin n.d.). This has been referred to as a “plasticity first” model. In contrast, plasticity can also be seen as an inhibitor of evolutionary change because fitness is disconnected from selectable genetic variation. The lack of extensive remodeling we observe here suggests that interspecific baculum divergence does not follow a “plasticity first” model, at least with respect to the mechanical stresses of intromission.

Our study provides comprehensive morphometric and high-resolution 3D imaging data, and suggests that mechanical loads associated with copulation are insufficient to drive strong changes in size, shape or composition. If copulatory interactions stress the baculum, they are not sufficiently intense or direct to induce adaptive remodeling.

## Data Availability

The data underlying this article are available in the Dryad Digital Repository, at https://doi.org/10.5061/dryad.jm63xsjrp
